# Steady as He Goes: At-Sea Movement of Adult Male Australian Sea Lions in a Dynamic Marine Environment

**DOI:** 10.1371/journal.pone.0074348

**Published:** 2013-09-25

**Authors:** Andrew D. Lowther, Robert G. Harcourt, Bradley Page, Simon D. Goldsworthy

**Affiliations:** 1 Threatened, Endangered and Protected Species, SARDI Aquatic Sciences, Adelaide, South Australia, Australia; 2 Department of Biological Sciences, Macquarie University, North Ryde, New South Wales, Australia; 3 Science, Monitoring and Knowledge Branch, Department of Environment, Water and Natural Resources, Adelaide, South Australia, Australia; Institut Pluridisciplinaire Hubert Curien, France

## Abstract

The southern coastline of Australia forms part of the worlds' only northern boundary current system. The Bonney Upwelling occurs every austral summer along the south-eastern South Australian coastline, a region that hosts over 80% of the worlds population of an endangered endemic otariid, the Australian sea lion. We present the first data on the movement characteristics and foraging behaviour of adult male Australian sea lions across their South Australian range. Synthesizing telemetric, oceanographic and isotopic datasets collected from seven individuals enabled us to characterise individual foraging behaviour over an approximate two year time period. Data suggested seasonal variability in stable carbon and nitrogen isotopes that could not be otherwise explained by changes in animal movement patterns. Similarly, animals did not change their foraging patterns despite fine-scale spatial and temporal variability of the upwelling event. Individual males tended to return to the same colony at which they were tagged and utilized the same at-sea regions for foraging irrespective of oceanographic conditions or time of year. Our study contrasts current general assumptions that male otariid life history strategies should result in greater dispersal, with adult male Australian sea lions displaying central place foraging behaviour similar to males of other otariid species in the region.

## Introduction

The distribution and foraging patterns of marine mammal top predators are influenced by bottom-up processes affecting lower trophic levels, such as oceanic currents and coastal upwelling events. These predators use three broad foraging strategies in response to the challenges of locating sufficient food to survive and reproduce in changing environments: epipelagic foragers such as sub-Antarctic fur seals *Arctocephalus tropicalis* [1] focus foraging efforts in the upper water column to depths of 200 m, while mesopelagic depths (200–2000 m) are the realm of predators such as the northern and southern elephant seals *Miroungia angustirostris* and *M. leonina* [2], [3] and benthic foragers such as Weddell seals [4], southern sea lions *Otaria flavescens* [5] and New Zealand sea lions *Phocarctos hookeri* feed on the sea floor [6]. Typically, epipelagic and mesopelagic foragers rely on predictable dynamic oceanographic features such as frontal zones and upwellings where enhanced productivity supports higher densities of prey [7], [8]. This imparts a degree of seasonality to foraging behaviour and this has been observed in a wide variety of predators including king penguins *Aptenodytes paptagonicus* [9], southern elephant seals [10], New Zealand fur seals *A. forsteri* [11] and sub-Antarctic fur seals [12]. As postulated by the ‘meeting point’ hypothesis, benthic or epibenthic prey tend to congregate around features such as shelf-breaks and reef systems [13], [14], [15], making such features important to benthic predators such as yellow-eyed penguins *Megadyptes antipodes* [16] and Australian fur seals *A. pusillus doriferus* [17].

Investigating the foraging behaviour of air-breathing marine predators typically involves the use of expensive geospatial tracking devices to describe movements of large, wild, elusive predators during foraging trips to sea. Consequently, prohibitive costs and logistical difficulties often limits answering questions of seasonal and intersexual differences in foraging to those that may be answered using data from a small subsample of animals [18], [19].Researchers face a further challenge when working with marine mammal predators such as otariid seals that undergo seasonal molts, as this restricts when tracking devices can be attached thereby defining the onset and duration of the temporal window over which tracking can occur. This limits the conclusions to be drawn on foraging behaviour to the specific temporal window over which tracking occurred. Otariids are a polygynous, sexually dimorphic order with adult males weighing two to four times the mass of adult females [20]. Female otariids have relatively long lactation periods, ranging from four to 36 months which constrain foraging to within reach of their dependent offspring [21]. Adult males, free from the constraints of providing parental care, should be able to forage across a wider range to ensure they can meet their greater size-related energetic costs [22].

Our understanding of the foraging behaviour of adult male otariids comes from limited studies of only seven of the 16 extant species. California sea lion adult males display both benthic and pelagic foraging behaviour, leaving the Gulf of California at the end of the breeding season to migrate north as far as Oregon into more productive waters before returning late spring with the onset of upwelling [22], [23]. Male Southern sea lions *Otaria flavescens* in South America also exhibit benthic and pelagic foraging behaviour, travelling twice the distance covered by conspecific adult females [24]. Pelagically-foraging male Antarctic fur seals *A. gazella* display post-breeding seasonal migration into (presumably) richer foraging grounds ∼900 km from their breeding colonies on South Georgia [25] whereas male northern fur seals *Callorhinus ursinus* move from shelf to oceanic waters outside the breeding season but do not travel as far as conspecific adult females [26]. In Australia, limited dive data suggests adult male Australian fur seals forage benthically [27] and show considerable individual variability in post-breeding foraging behaviour, with some individuals remaining in the same foraging grounds utilized by adult females and others moving considerable distances [28]. Further west, adult male New Zealand fur seals *A. forsteri* in South Australia feed along a shelf-break and their dives are typically pelagic, but approximately 20% of their dives are benthic [29], [30]. In all of these studies, conclusions on the temporal variability in foraging behaviour have been limited by the battery longevity of telemetry devices and how long they can remain attached.

The use of cost-effective stable isotope biogeochemistry has provided greater insights into foraging ecology across a range of marine mammals [31]. There is strong support for the upward-cascade of stable nitrogen isotopes (δ^15^N) from primary producers to the top of the food web. In marine ecosystems a decreasing stable carbon ratio (δ^13^C) gradient has been identified with distance from shore [32], [33]. Isotope ratios provide ecological geotrophic data that can be used to describe temporal and individual variation in foraging location and diet outside the scope of traditional tracking methods, using δ^13^C and δ^15^N from metabolically active (blood) and inert (whiskers, feathers etc) tissues in several seal and marine bird species [34], [35], [36], [37], [38]. Multi-year, individual variation in foraging behaviour has been described using isotope values from serially-subsampled whiskers of adult male Antarctic fur seals [39] and adult female Australian sea lions *Neophoca cinerea* [40].

**Figure 1:**
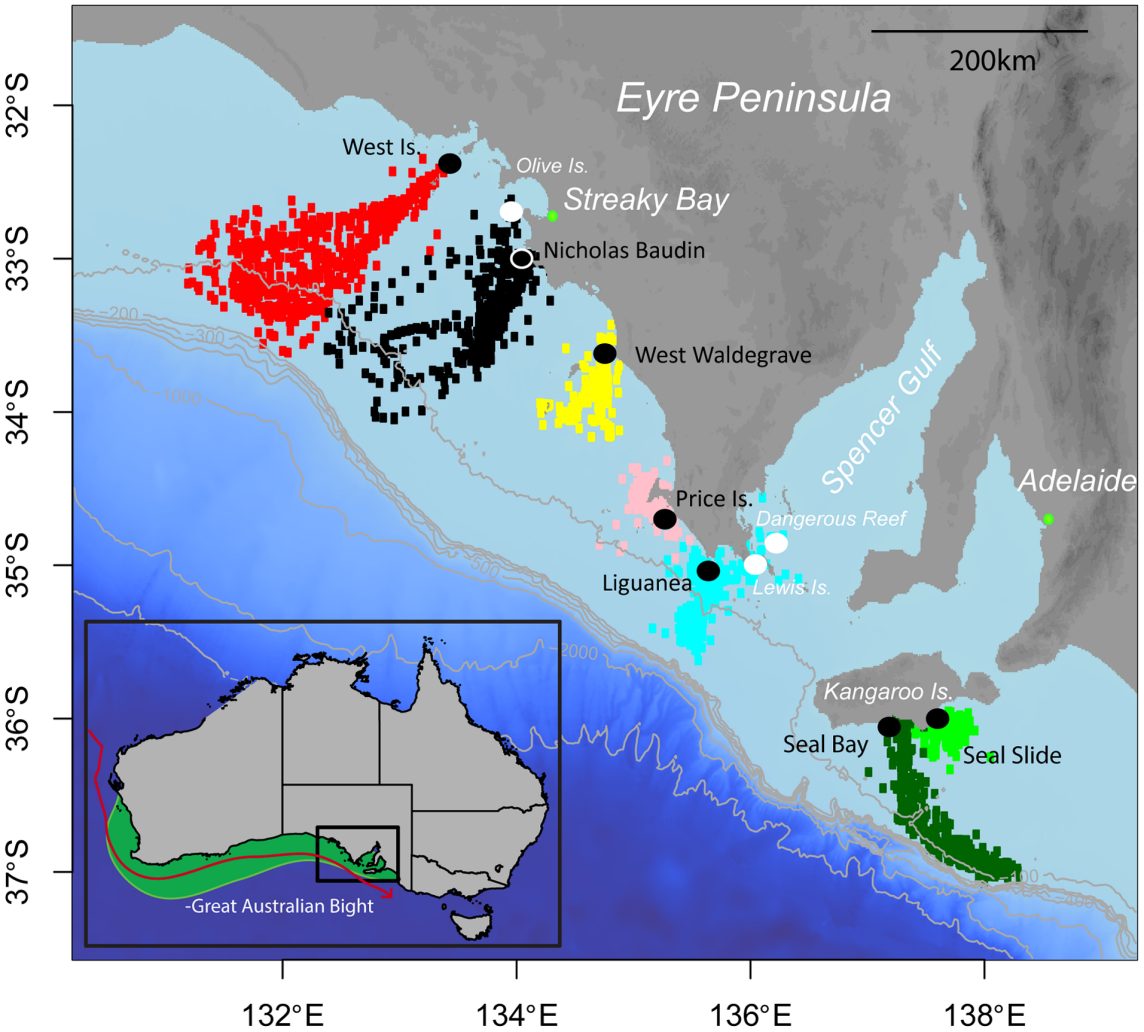
Location of study sites relative to Australian sea lion species distribution. **(Inset)** The endemic Australian sea lion is distributed from Kangaroo Island in South Australia to the Abrohlos Islands in Western Australia (Inset – light green relief). A seasonal cold-water upwelling event occurs of the southeast coast of Kangaroo island during the austral spring/summer, permeating westwards until the onset of the eastward-flowing Leeuwin Current during autumn/early winter (red line depicting Leeuwin Current). (**Main Figure**) Telemetric, oceanographic and isotopic data were collected from individual male Australian sea lions at seven breeding colonies (black circles, black text) throughout the South Australian range, encompassing 80% of the worlds' population. Bathymetry shown incrementally to 3000 m. Filtered, interpolated two-hourly locations covering the entire tracking period are colour-coded for each individual. There were no obvious changes in movement behaviour by individuals throughout the period.

The biogeography and unique reproductive ecology of the Australian sea lion has made characterising its foraging ecology challenging. This endangered species [41] has a census estimate of ∼15,000 animals making it one of the rarest otariids in the world. Australian sea lions are distributed from the Pages islands in South Australia to the Abrolhos Islands in Western Australia with 85% of pup production occurring within South Australian waters [42] (Figure 1). A protracted, aseasonal breeding pattern (∼17.5 mo) that is temporally asynchronous across its range is unique amongst pinnipeds where annual synchronous breeding is the norm [43]. The lack of synchrony to breeding is intriguing given the highly seasonal breeding patterns of Australia's other two resident otariids, the Australian and New Zealand fur seals. The fact that adult females display long-term fine-scale foraging site fidelity further suggests that there are adequate resources available year-round [44].

The hypothesis that Australian sea lion life history and foraging strategies reflect a species adapted to an oligotrophic, aseasonal marine environment has been suggested [44]. However, a seasonal cold water upwelling system along the South Australian coastline attracts large aggregations of cetaceans annually during the austral summer [45]. Furthermore, the region is occupied by the largest and fastest-growing breeding colonies of New Zealand fur seals in Australia [46] and supports some of the largest volume commercial fisheries in the country. Rather than being an aseasonal environment, the region appears to be replete with mesoscale areas of primary productivity and seasonality. Although upwellings and associated frontal systems tend to attract pelagic predators, the presence of the largest Australian sea lion breeding colonies of the species in the nearby vicinity suggests, indirectly at least, upwellings also create conditions suitable for benthic foragers. The advent of animal-borne sensors of the same quality as those used by oceanographers provides previously unavailable high-resolution oceanographic datasets along individual animal trajectories [47]. In light of the unique reproductive ecology of Australian sea lions and complete absence of information on adult male Australian sea lion at-sea movement, we employed a combination of telemetric, oceanographic and biogeochemical techniques similar to those successfully used on conspecific adult females [40]. Specifically we wished to determine 1) the temporal and spatial extent of the regions' seasonal upwelling system, 2) whether individuals altered their at-sea behaviour in relation to changing environmental conditions and 3) the degree of individual variability and temporal consistency of foraging behaviours.

## Materials and Methods

### Study sites and sampling

Movement and environmental data were collected using oceanographic sensors deployed on a single adult male Australian sea lion from each of seven breeding colonies spanning the South Australian range between October 2009 and June 2010 (Figure 1). Males were initially sedated using zolazepam-tilamine (300–350 mg Zoletil® Virbac Ltd) delivered remotely by a NO_2_-powered tranquilizer gun (Taipan, Tranquil Arms Ltd). Once immobilized, animals were anaesthetized using isoflurane delivered through a portable gas anesthesia machine (5% induction, 1–3% maintenance; Veterinary Companies of Australia). A Conductivity-Temperature-Depth Satellite-Relay Data Logger (CTD-SRDL; Sea Mammal Research Unit Ltd, Scotland) weighing ∼540 g was attached to the pelage distal to the midpoint of the back of each individual using two-part epoxy glue (Araldite® K-268). Prior to recovery from anesthesia procedure a whisker was clipped at the base of the muzzle from each individual for isotopic analysis.

### CTD-SRDL sampling protocols

The sensor platform within the CTD-SRDL comprised a Keller PA-7 pressure transducer (accuracy 2±0.3DBar), a custom-made temperature sensor (resolution 0.001°C, accuracy ±0.005°C) and an inductive coil to measure conductivity (resolution 0.002 mS/cm, accuracy ±0.01 mS/cm). Instruments were calibrated after assembly [47] and CTD profiles collected during dives were transmitted via the ARGOS satellite network. Due to bandwidth constraints of the ARGOS network, not all the collected profiles could be transmitted. For a detailed overview of the instruments, onboard data handling, compression, transmission, location and dive depth estimation see [47].

### Vibrissae preparation and isotopic analysis

All vibrissae were cleaned as described in [38]. Available data on otariid whisker growth rates suggest linear growth at a rate of approximately 3 mm/month [39], [48]. We use this growth rate as a general guide to sample monthly isotopic values indicative of geotrophic behaviour, similar to that performed by [40], acknowledging that the growth rate is estimated in the absence of species-specific verification. Vibrissae were cut into 3 mm segments starting at the base and placed in labelled 7 ml glass scintillation vials. All samples for isotope analysis were analysed at the Australian National University Environmental Biology Stable Isotope Facility. Analysis was performed using a Microass isoChrom CFIR mass spectrometer coupled to a Carlo Erba EA-1100 CHN-O analyser. The standard control ratio for ^13^C and ^15^N were Pee Dee Belemnite and atmospheric nitrogen, respectively. Observed measurement error rates were recorded as <±0.1‰ (δ^13^C) and <±0.3‰ (δ^15^N).

## Analysis

### Location data processing

Raw ARGOS data were pre-processed to remove extreme outliers by removing locations with unclassified error estimates (LC- Z) and by using a swim-speed filter with a moving average of 2 ms^−1^ [49]. Further filtering involved estimating locations using a Kalman filter under a state-space framework in the R package ‘crawl’ [50]. A foraging trip was defined as continuous periods at-sea greater than 6 h duration [51] and its initial state (location) identified when the instrument entered or exited haulout mode (triggered by an onboard saltwater switch). Using these definitions, location records for each animal were partitioned into foraging trips for which duration (d) and distance travelled (total horizontal Great Circle distance, km) were calculated.

### Habitat utilization

To quantify space-use and the environment the animal experienced while foraging, two parameters were derived for each foraging trip. First Passage Time (FPT) was used to identify the scale at which Area Restricted Search (ARS; i.e. inferring active foraging behaviour) was conducted [52], [53]. To perform FPT, each track was redescretized into 1 km step lengths and a new time index interpolated for each new position. The FPT of circle radii ranging from 0.5 km to twice the maximum daily step length [54] were calculated and the radii responsible for varlog_max_FPT identified for each animal (herein referred to as ARS patch size). FPT values for this radii were then plotted against time for each track, with ARS behaviour being inferred at segments of tracks that took the longest time to traverse (i.e. expressed maximum FPT values) [55], [56], [57]. The second set of metrics calculated were the utilization distributions (95% trip range and 50% core range, km^2^) for each trip of each individual, using a Brownian Bridge movement model (BBMM) described in [58] to account for serial autocorrelation in location data implemented in the R package ‘adehabitatHR’ [59]. Static parameters (depth of the sea floor (m) and bathymetric slope (°)) were derived from a 9 arc-second (250 m) resolution bathymetric digital elevation model of the region [60] and values for each metric were extracted for each FPT location using R.

### Spatio-temporal variability in oceanography

Surface mixed layer depth (MLD) can be defined in terms of a critical density gradient or by a finite change in density from surface values [61]. Given the coarse nature of depth sampling provided by compressing Temperature-Salinity (T-S) profiles we employed the latter [62], calculating seawater potential density anomalies (σ ⊖, kg m^−3^) at one metre intervals for each CTD profile using the Thermodynamic Equation of State of seawater [63]. To avoid aliasing issues with the diurnal heating-cooling cycle and to acknowledge the effect of surface turbulence, we use a conservative value of 0.125 kg m^−3^ and define the surface σ ⊖ as the value calculated for the bottom of the first depth bin (4 m) to determine MLD at the location of each CTD profile [61], [64], [65]. Monthly means of temperature and salinity at 1 m depth and at the sea floor (determined as being represented by the maximum depth bin of each CTD profile – see below), thermocline intensity (maximum change in temperature per metre, °C m^−1^) and MLD (m) were calculated and interpolated across the study area on a 1 km resolution grid using ODV4.5.1 [66] and R. Parameter values were then extracted along each track at locations with FPT values.

### Spatio-temporal variation in foraging behaviour

Prior to modelling, a coarse characterisation of dive behaviour was made by regressing maximum dive depth on extracted mean water depth for each individual (regression F>91.2, R^2^ >0.83, p<0.001 in all cases). This exploratory process indicated adult male Australian sea lions exhibited a benthic diving strategy, suggesting environmental conditions at the surface may have been unimportant to foraging. Accordingly, in conjunction with static features (bathymetric slope and depth), we considered only temperature and salinity at the sea floor, MLD, transition layer thickness (the depth between the bottom of the MLD and the sea floor) and thermocline intensity as biologically-relevant, dynamic variables. The significance of temporal variability in core (UD-50) and home (UD-95) ranges, both between individuals and among trips for each individual, were characterised using Kruskal Wallis Χ^2^. Subsequently, for those individuals that expressed significant variability, we examine the influence of environmental conditions on individual core, home and ARS patch sizes using generalised additive mixed models (GAMM in R package ‘mgcv’). Accounting for the expected high degree of correlation between most oceanographic variables (Pearsons Product Moment Correlation t>36.5, p<0.001 for all significant cases), models ranged in complexity from null models to the inclusion of all uncorrelated covariates (and their interactions). However all models included month-since-deployment as a fixed factor and individual foraging trip as a random factor (Table S1). Penalised regression splines for each parameter were employed in all GAMM's and optimised by restricted maximum likelihood (REML) methods [67]. ANOVA's were used to check for correlation of residuals against fitted values and confirm the appropriateness of candidate models (those which contained significant explanatory terms). The optimal model for each individual was then identified by a likelihood ratio test and confirmed using Bayesian Information Criteria.

We tested the effect of environmental variability on FPT using a series of individual Cox Proportional Hazard models (CPH) of the form: *h*(*t*)  =  exp(β_1_ *X*_1_ +β_2_ *X*_2_ +β_3_ *X*_3_ +...+β_p_ *X*_p_)*h*_0_(*t*), where h(t) is the risk (hazard) of an individual leaving an area (defined by the FPT across a predetermined radius) at time *t*, X represents a physical variable (bathymetry, slope, rugosity), β denotes the regression coefficient fitted to each variable during the modelling process and *h_0_(t)* the baseline hazard function (the risk of leaving an area when all explanatory variables equal zero). In this context, a hazard ratio provides a quantitative means to assess how an animal responds to changes in environmental parameters [68], [69]. For a more comprehensive review of the technique and the manner in which results are interpreted, see [70]. Similar to the GAMM process, all possible combinations of (uncorrelated) environmental parameters and their interactions including null models were fitted during model selection. Optimal model selection (from those models which had significant explanatory terms) was performed using Akaike Information Criteria corrected for effective sample size (AIC_c_) [70]. All CPH models were fitted in R using the ‘survival’ package.

### Variation in whisker isotopes

Temporal variation in foraging behaviour was quantified using univariate ARIMA (*p,d,q*) (AutoRegressive Integrated Moving Average) models to stable carbon (δ^13^C) and nitrogen (δ^15^N) isotope values implemented by a three-step Box Jenkins method [71]. ARIMA (*p,d,q*) models require the estimation of the number of autoregressive parameters (*p*), the differencing order (*d*) and the variance of the error term (‘moving average’) parameters (*q*). When data exploration suggested seasonality, additional parameters in the form (*p,d,q*)*_s_* required estimation with ‘s’ representing the periodicity of seasonal influence. Thus, the minimum-parameter first order Seasonal ARIMA(1,0,0)(0,0,0)_0_ is essentially an aseasonal first-order autoregression that can be characterised as a temporally-correlated random walk, with the current value being influenced only by the value immediately preceding it. Conditional sum of squares (to assess initial parameter values) followed by exact maximum likelihood methods were employed to estimate each parameter [72]. Finally, models were validated by checking for autocorrelation of residuals using autocorrelograms and portmanteau tests (Ljung-Box Q statistic) [73]. Optimal ARIMA models were then selected using Bayesian Information Criteria (BIC) as this method penalises overfitting and is widely used in time series analyses [74].

## Results

### Movement behaviour

Between November 2009 and May 2010 CTD-SRDL datasets were collected from seven adult male Australian sea lions along the South Australian range of the species (Figure 1). Transmitters remained attached for an average 161d (±13) (range 99–213 d), recording 1486 (±528) locations per individual. Post-processing and filtering left approximately 1182 (±248) locations each. Adult males made a mean 28 (±3) foraging trips (range 19–42) described by approximately 49.5 (±9.46) location fixes per trip. Foraging trips were an average 3.6d (±0.49) in duration (range 0.5–7.1 d) with animals covering a mean 167.9 (±33.26) km (range 7.2–368 km) per trip (Table 1). Individuals dived to a mean depth of 62.2 m (±9.46 m) with mean and maximum dive depth ranges of 15–86 m and 50–137 m, respectively (Table 1). The conductivity sensor failed on one transmitter (Seal Slide), which was then excluded from all subsequent oceanographic and habitat preference analyses. The remaining six individuals collected an average 897 (±68) CTD profiles each (range 594–1125) throughout the tracking period, at a mean rate of 38.2 (±12.6) profiles per foraging trip (range 26–51). Animals that travelled the furthest (West Island and Seal Bay) had the largest ARS patch size, core and home ranges while the individual at Seal Slide had a mean home range less than 200 km^2^ (Table S1).

**Table 1:** Deployment and foraging trip data from seven adult male Australian sea lions instrumented between November and December 2009 with satellite-linked Conductivity, Temperature and Depth (CTD) loggers.

Animal ID	Instrumentation date	Duration (d)		Trips							Dive depth (m)	
		Total	At-sea	N	Distance (km)		Duration (d)		Speed (km h^−1^)			
					mean (sd)	range	mean (sd)	range	mean (sd)	range	mean (sd)	max
**West Is.**	11-Dec-09	160.5	106.3	19	350.9 (44.5)	254.2–446.6	5.6 (0.8)	4.2–7.7	2.6 (0.4)	2–3.2	86.3 (19.4)	137.5
**Nicholas Baudin**	08-Dec-09	178.6	120.3	29	216.3 (73.9)	127.2–417.2	4.1 (1.2)	1.6–7.3	2.2 (0.5)	1.5–3.3	65.7 (16.5)	101.5
**West Waldegrave**	10-Dec-09	205.7	110	42	113.5 (52.1)	10.7–309.3	2.6 (1.3)	1.1–9	1.9 (0.7)	0.2–4.2	55.7 (15.7)	88.5
**Price Is.**	28-Nov-09	213.4	51.8	33	43.4 (32.9)	15–107.4	1.6 (1)	0.5–3.9	1.2 (1.1)	0.01–6.6	15.2 (7.13)	50.5
**Liguanea**	29-Nov-09	99.1	52.8	25	82.1 (62.1)	13.1–198.9	2.1(1.6)	0.3–5.4	1.7 (0.8)	0.6–3.6	83.9 (32.1)	129.5
**Seal Bay**	03-Dec-09	175.1	123.8	23	299.8 (33.9)	237.3–368.1	5.4 (0.6)	4.3–7.1	2.3 (0.3)	1.8–2.7	79.2 (18.6)	105.3
**Seal Slide**	07-Nov-09	179.9	96.8	27	69.7 (26.31)	7.2–134.4	3.6 (0.8)	1.5–4.6	1 (0.3)	0.2–1.8	49.7 (14.5)	68.5

Deployment duration was typically in excess of five months with the exception of the individual at Liguanea which ceased transmitting after little over three months. Foraging trips ranged from 0.3–7.7 d and 7.2–446.6 km in duration and distance, respectively, conducting between 19 and 42 foraging trips each. Most animals spent between 50–70% of their respective tracking periods at sea. The male from West Island achieved the greatest maximum dive depth, while the individual from Price Island reached a mean depth of 15.2 m and spent less than 25% of its tracking period at sea.

### Spatio-temporal variability in the oceanography of the eastern Great Australian Bight

**Figure 2:**
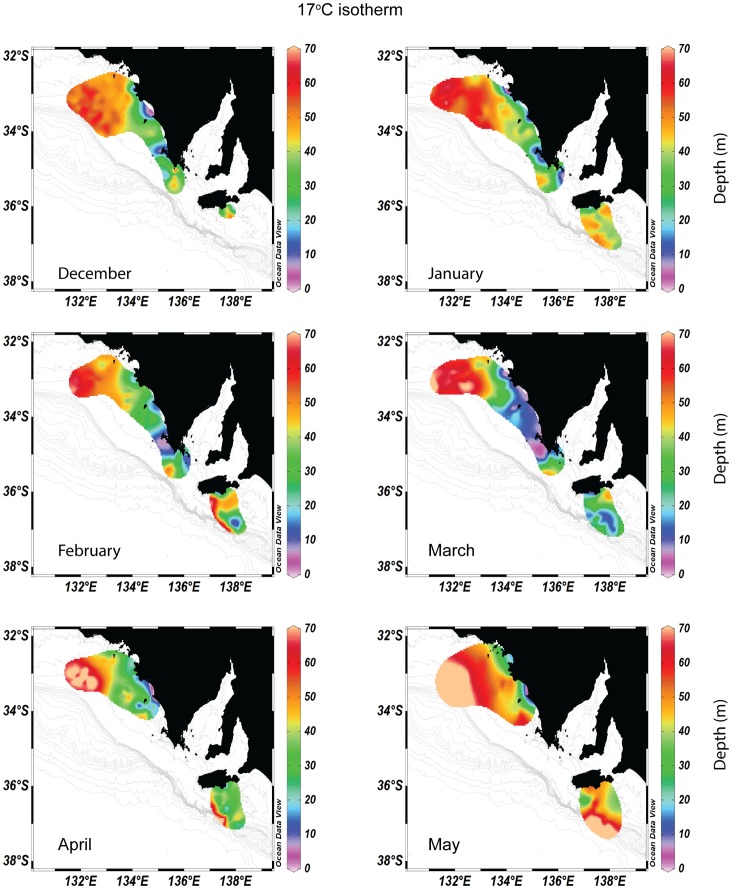
Depiction of cold water upwelling along the South Australian coast as a function of the depth of the 17°C isotherm across the study region between December 2009 and May 2010. Temperature profile data from Conductivity, Temperature and Depth (CTD) tags deployed on seven adult male Australian sea lions during the austral summer upwelling event in 2010. The 17°C isotherm was detected in relatively shallow waters (<40 m) as late as April throughout the geographical range of the study. Wind-trapped pockets of cold water were consistently detected at the surface at Price Island and West Waldegrave throughout most of the study.

**Figure 3:**
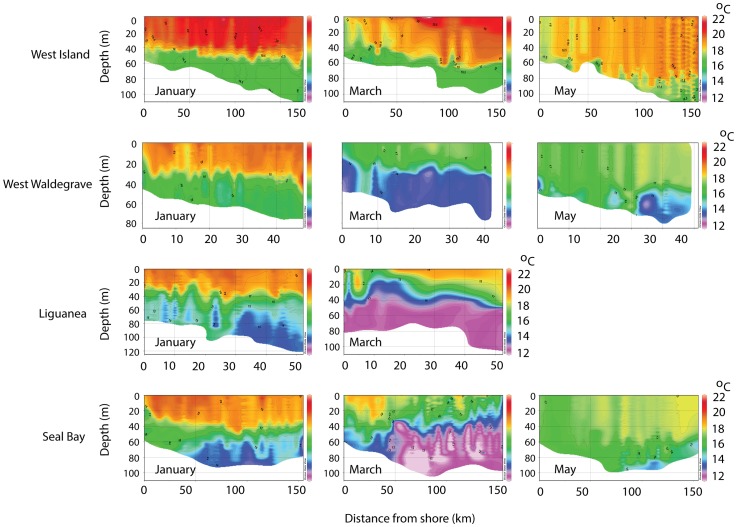
Temporal changes in water column thermal structure throughout the study range derived from CTD profile data. Water depth (m) and perpendicular distance from shore (km) are represented on the X and Y axes, respectively. Cross-sectional water temperature profiles demonstrate thermal stratification both at and among sites, with the onset of the eastward-flowing Leeuwin Current leading to almost complete mixing by May and signifying the end of summer upwelling. Truncation of the data from the Liguanea male is due to the CTD ceasing transmission at the end of March. Similar variability in mixed layer and haline structuring was observed over the same period (not shown). The reduced number of sites and bi-monthly representation are intended purely to reduce figure complexity while still capturing the temporal and geographical extent of oceanographic variability.

Using the threshold criteria of [75] for defining upwelled water (temperature:<17°C, σ ⊖>26 kg m^−3^, salinity:<35.6 kg m^−3^), upwelling occurred between December and March along the western Eyre Peninsula reaching as far as Cape Bauer near Streaky Bay (Figure 2). Upwelled water also reached the surface consistently between Coffin Bay and Elliston over the same period, being most evident during March (Figure 2). Cross-sectional visualization of the water structure also revealed substantial variation over the region (Figure 3). Although the upwelling plume did not reach the surface at the western extent of the study region, it was detectable at West Island as shallow as 30 m in March. In all cases there was diminished thermal stratification of the water column towards the start of the Austral winter (May) signifying the end of the upwelling season (Figure 3). Between January and May, individuals experienced significantly different monthly mean benthic temperatures and salinities (between-individuals in the same month; Kruskal-Wallis X^2^>13.7, p<0.001). Mean benthic conditions also varied significantly for each individual throughout their respective tracking period with the exception of West Island (between-months for the same individual; Kruskal-Wallis X^2^>14.1, p<0.001 in all significant cases; Figures 3). There were positive relationships between mixed layer depth and transition layer depth with ocean depth (mixed layer depth: regression F_1,185_ = 88, p<0.001, R^2^ = 0.33; transition layer depth: regression F_1,185_ = 641, p<0.001, R^2^ = 0.78). Thus, when upwelling was present, individuals that foraged in the deepest water experienced the thickest mixed layer and thickest transition layer (ANOVA F>4.6, p<0.05 in all cases).

### Spatio-temporal variability of habitat use by individuals

Generally, individual movement patterns did not change throughout the study period (Figure 1). However, the male at Nicholas Baudin did move between its tagging site and nearby Olive Island on several occasions though at-sea movements tended to be in the same general areas. Similarly the Liguanea male also moved between colonies, visiting Lewis Island in the Thorny Passage and Dangerous Reef in the southern Spencer Gulf on several occasions (Figure 1). Only males from West Is, Nicholas Baudin and Seal Bay displayed significant relationships between areal metrics and any environmental variables (Table S1). The male at West Island decreased both core and home ranges in response to rising benthic salinity (GAMM F_smooth(benthic salinity)_ = 7.6 and 5.1, respectively, P<0.05 in both cases). Conversely, despite exploiting at-sea regions close to those used by the West Island male, the individual at Nicholas Baudin did not appear to respond to changes in dynamic features, instead decreasing its core range at shallower depths (GAMM F_smooth(bathymetry)_ = 44.6, P<0.001) and steeper slopes (GAMM F_smooth(bathymetry)_ = 3.5, P<0.05). At Seal Bay the male started to increase its core range when bottom temperatures started to rise above 13°C (GAMM F_smooth(benthic temperature)_ = 6.9, P<0.001; Table S2).

In terms of residency times along individual tracks, optimal CPH models failed to include any dynamic variable for any individual except for the West Island male, which showed an aversion to warmer bottom temperatures (Table S3). The male at West Waldegrave expressed a general preference for habitat in shallower water, decreasing residency times by ∼10% with each metre of depth (Table S3). The Liguanea individual displayed a preference for flat terrain, displaying a strong aversion to bathymetric slope. For both males these patterns were consistent over time, with a low spread of per-trip variability in the risk of leaving (∼17% in both cases). Variability in FPT of the males at Nicholas Baudin, Price Is. and Seal Bay were parsimoniously characterised by a null model (Table S3).

### Isotopic variability

**Figure 4:**
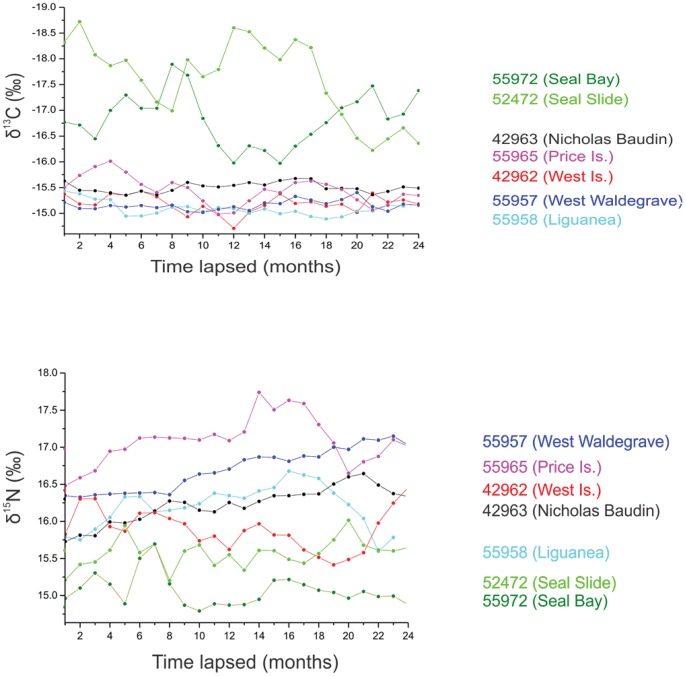
Temporal variation in stable nitrogen (δ^15^N – upper) and stable carbon (δ^13^C – lower) isotopes along the whiskers of individual male Australian sea lions over an estimated two year time period. Individual δ^15^N and δ^13^C enrichment generally increased westwards. Significant seasonal patterns were detected in both δ^13^C and δ^15^N in all individuals, however seasonality in δ^13^C was considerably stronger at the eastern end of the study (Seal Bay and Seal Slide) than at any other site.

Individual values for δ^13^C and δ^15^N ranged from -14.7 to -18.2‰ and 14.8 to 17.7‰, respectively. Mean carbon and nitrogen isotope ratios generally increased along a longitudinal cline (Kruskal Χ^2^>161.8, p<0.001 in both cases; Table 2). The adult male at Seal Bay displayed the lowest mean carbon ratios of all colonies (−17.6±0.16‰), followed by Liguanea (-16.8±0.1‰) (Students t-test t>15.6, p<0.001 in all cases; Table 2). The notable exception in mean nitrogen values was the individual from Liguanea, which had the lowest ratios detected (15±0.04‰), more than 2‰ below the highest recorded mean value (West Island, 17.1±0.06‰,) (Students t-test, t = 25.7, p<0.001). Individual δ^13^C and δ^15^N variability (where present) were best characterised by first-order ARIMA models with a seasonal periodicity ranging from 3–12 months (Table S4, Figure 4). Significant seasonal patterns (in terms of variance of carbon isotope ratios) were detected for all animals but the magnitude of seasonality differed significantly between individuals (Bartletts test, K = 174, p<0.001; Figure 4). Variance around mean carbon ratios for individuals at Seal Bay and Seal Slide (0.6‰ and 0.26‰, respectively) were a minimum of an order of magnitude greater than all other individuals (Table 2).

**Table 2:** Stable carbon (δ^13^C) and nitrogen (δ^15^N) isotope data from whiskers of seven adult male Australian sea lions sampled along the South Australian coastline.

Animal ID	Instrumentation date	δ^13^C (‰)			δ^15^N (‰)		
		mean	variance	range	mean	variance	range
**West Is.**	11-Dec-09	−15.5	0.07	−16–15	17.1	0.10	16.5–17.7
**Nicholas Baudin**	08-Dec-09	−15.5	0.01	−15.7–15.4	16.2	0.06	15.7–16.6
**West Waldegrave**	10-Dec-09	−15.2	0.01	−15.4–15	16.7	0.08	16.3–17.1
**Price Is.**	28-Nov-09	−15.1	0.01	−15.4–14.9	16.2	0.08	15.6–16.6
**Liguanea**	29-Nov-09	−15.2	0.08	−15.4–14.7	15.9	0.08	15.4–16.4
**Seal Slide**	03-Dec-09	−17.6	***0.60***	−18.7–16.2	15.5	0.04	15.2–16
**Seal Bay**	07-Nov-09	−16.8	***0.26***	−17.9–16	15.0	0.08	14.8–15.7

Mean, variance and ranges (‰) shown for each animal. δ^15^N generally showed a distinction between animals east and west of Spencer Gulf/137°longitude. Individuals at Seal Slide and Seal Bay displayed variance in δ^13^C (in bold) approaching an order of magnitude greater than any other individual.

## Discussion

We present the first comprehensive study of adult male Australian sea lion foraging ecology, identifying long-term individual fidelity to geographical areas and substantial inter-individual variability in foraging habitat selectivity throughout the formation and decline of a seasonal upwelling system. Integrated biogeochemical, telemetric and oceanographic data provides a powerful means of exploring the interactions between a wide-ranging marine predator and its environment in the context of oceanographic variability. Given the strong positive relationship between maximum dive depth and bathymetry, we conclude that adult male Australian sea lions exhibit a benthic foraging strategy. Whether they are obligate benthic foragers is unknown. Adult male Australian sea lions in South Australia display minimal reliance on dynamic environmental variation typified by seasonal upwelling. The study spanned one of the most intense upwelling events in South Australian history and was measured *in situ* by the animals themselves, thus we are confident our data appropriately reflect the response of adult males to dynamic oceanography. Some individuals conducted cross-shelf foraging trips perpendicular to the dominant oceanographic currents in the region but others occupied foraging grounds much closer to the colonies at which they were instrumented. The existence of summer upwelling along the southern coastline of Australia has been known for over 40 years [76]. Previous studies of upwelling dynamics throughout the region have been based on limited in-situ data, conceptual models or on studies conducted over a short time period (4–6 weeks) [75], [77]. Fitting adult male Australian sea lions with oceanographic sensors has enabled us to collect data describing the four-dimensional spatial extent of a seasonal upwelling event in southern Australia over a six-month period. We show that adult male Australian sea lions may be a tractable group for future study of biological and physical oceanographic processes in this unique northern boundary current system.

### Foraging behaviour unaffected by dynamic oceanographic processes

Trophic structure and function in marine ecosystems is a result of the interaction between the dynamic features of physical ocean processes and the structure of the benthos [78]. A lag between the onset of physical ocean processes and primary and secondary productivity often results in temporal and spatial variability in structure and composition of the foodweb throughout the water column [79]. It has been suggested that spatial de-coupling between physical oceanography and primary and secondary productivity may have been responsible for the lack of fine-scale relationships between Antarctic fur seal foraging behaviour and chlorophyll a [80]. The interactions between oceanic processes and the continental shelf can also influence community structures. Off the coast of Oregon, differences in current strength and upwelling intensity along an asymmetrical shelf result in greater food availability that persists outside the upwelling season at inshore communities along the widest sections of the shelf [79]. In California, larval euphausids spawned during winter upwelling reach adulthood approximately four months later, creating a bloom in zooplankton [81]. Adult euphausiids then track a shoreward collapse in oceanic productivity into the Monterey Bay at the end of the upwelling season, coinciding with the seasonal aggregation of blue whales [81], [82].

The eastern Great Australian Bight can also be characterised by an asymmetrical shelf structure increasing in width westwards and punctuated along its length by submarine canyons with the eastern end often described as the centre of seasonal upwelling [75], [77], [83]. Nutrient rich, carbon-depleted waters from the subtropical front (STF) pulse onto the shelf during the Austral summer in a series of upwelling events [77] forming what is referred to as the ‘Kangaroo Island pool’ [75]. Available literature on the upwelling process suggests that this pool represents the source of upwelled water along the eastern Great Australian Bight [75]. Subsequent wind-shear pulls water from this pool and drives it along the coastline of the western Eyre Peninsula throughout the Austral summer in a series of wind-trapped coastal upwelling events. Progressive mixing of upwelled water with more ^13^C-enriched coastal and shelf waters coupled with prolonged onshore winds may result in the pooling of nutrients along the wider western margins of the shelf in a process similar to that outlined by [78]. Further alteration of the carbon signal would occur with the introduction of warm, carbon-depleted Leeuwin Current water at the start of winter [84]. Our study supports this hypothesis, with seasonal variation in ^13^C appearing to decrease at sites west of Kangaroo Island reflecting the mixing of ^13^C-depleted waters from the upwelling site with more ^13^C rich coastal waters farther west. Interestingly, adult male Australian sea lions who displayed the lowest δ^13^C also fed consistently at the lowest trophic levels in the study. Interaction between topographical breaks in the shelf, longitudinal variation in the distribution of upwelled water, the intrusion of a tropical water mass into the region at the end of summer and the spatial decorrelation between the availability of nutrients and secondary productivity are likely to lead to variability in prey guild composition, age structure or a combination of both. These complex, fine-scale processes likely drive the patterns of isotopic markers we describe and explain why most adult male foraging behaviour did not track with dynamic changes in the physical environment.

### Spatial and temporal variation in isotopic signatures suggest foraging behaviour is temporally persistent

There are several assumptions implicit to the interpretation of our isotopic data. Foremost, we had no species-specific whisker growth rates thus our assumption of 3mm representing an isotopic timeline of one month may be incorrect. Furthermore, the possibility that individuals undergo periodic whisker replacement may also have confounded our interpretation. Empirical estimates of whisker growth rates in otariids are restricted to Steller sea lions and Antarctic fur seals [39], [48] with both studies suggesting whiskers grow consistently at similar rates (0.1–0.16 mm d^−1^) and are retained year-round, in contrast to phocid seals such as the gray seal and harbor seal [48], [85]. Thus we argue the context in which we interpret our isotopic data is likely to be robust to small errors in timeline estimation. Furthermore, the seasonal carbon cycles we detect at the eastern end of our study reflect the seasonal cycle of upwelling in the region. Importantly, the biogeochemical markers used in our study highlighted what appeared to be broad differences in the trophic ecology of individuals foraging towards the epicentre of the upwelling compared to those at its western extremity. Individuals at Seal Bay and Seal Slide displayed a discrete shift in δ^13^C, a pattern commonly interpreted in other otariid and seabird species as representing seasonal migration [34], [39], [86], [87]. Taken in isolation, the geotrophic data we present for these individuals may have been mistakenly interpreted as reflecting broad-scale differences in the foraging ecology of individual adult males, with eastern males undergoing seasonal changes in foraging location.

Our study is a cautionary tale about relying solely on biogeochemical markers to infer geotrophic behaviour. The incorporation of prolonged time-series telemetry data and information about oceanography enables us to interpret our isotopic data in a more appropriate manner. There were no consistent trends in adult male at-sea movement that related to dynamic oceanographic variability and the manner in which individuals explored their environment. Adult males in the region that experienced the greatest environmental variation (Kangaroo Island) did not alter their movement patterns throughout the entire upwelling season (Dec – May). Indeed, the individual who foraged on the shelf break consistently occupied similar habitat to those recorded for adult male New Zealand fur seals [30]. The lack of seasonal foraging patterns in adult male Australian sea lions and New Zealand fur seals suggests there is adequate, suitable prey available all year at or near the benthos though the strong seasonality of the marine environment in the region may result in a more diverse, seasonally-variable suite of prey items. The strong seasonal signal in ^13^C and ^15^N detected in these eastern individuals probably reflects seasonal changes in the structure of prey guilds rather than changes in where individuals foraged. Thus we strongly recommend employing biotelemetry devices to put isotopic data into context, particularly in temperate latitudes or coastal marine environments where isoscapes are poorly defined or highly variable. Assuming our interpretation is correct, we also caution against generalising adult male Australian sea lion diet based on limited temporal or spatial sampling. The apparent long-term fidelity of adult male Australian sea lions to individual colonies we show does provide an opportunity to generate species-specific whisker growth rates. Biological markers such as isotopically-labelled glycine could be administered to individuals during the initial deployment of transmitters, creating a highly-visible marker that would be evident in whiskers removed at the end of a deployment [48].

### Individual variation in foraging movement

If suitable prey exists year-round along the South Australian coastline then why do males not forage more broadly, particularly given they are not restricted by the need to provide parental care? Even though some individuals in our study could conceivably reach the same foraging grounds as males from neighboring colonies, they did not do so. This suggests that the parameters we measured were not sufficient to explain strong individual preferences for particular foraging habitat. However, tracking one male at each colony hampers our ability to generalise any behaviour. For example we do not know whether the strategies adopted by individuals we followed are representative of all males at a colony, or are one of several behaviours within a colony [40]. The former hypothesis points towards sufficiently strong local ecological and environmental pressure to influence all the males resident at each colony, whereas the latter may indicate strong individual experiences of known features associated with consistent prey [13]. Coastal upwelling was most persistent around the lower western Eyre Peninsula, where individuals at Price Island and West Waldegrave remained foraging relatively close to shore. These explanatory factors are not likely to be mutually exclusive; males may be able to access a preferred foraging location from several nearby colonies, unlike those at other more isolated colonies. Thus we recommend additional tracking to test these hypotheses and elucidate further on the influence of oceanographic variability at a colony-level. An inability to link environmental parameters with foraging activity point towards information missing from our analyses. A notable gap in our data is the availability and diversity of prey, which should be filled by direct sampling throughout the region. Furthermore, additional telemetric studies on adult male Australian sea lions should incorporate measures of individual internal condition such as age or body mass that may influence the inter-individual variability in strategies we describe.

Our interpretations may be confounded by assuming that the location at which a male was initially captured represents its home colony. On more than one occasion the Nicholas Baudin male hauled out at nearby Olive Island, the fifth largest breeding colony of the species that at the time of instrumentation was over two months past a breeding episode. Conversely, the Liguanea male made several trips up the Thorny Passage into the southern Spencer Gulf, stopping at two colonies of which one was entering a breeding cycle during the tracking period (Lewis Island, Figure 1). This individual was observed at the breeding colony on two separate occasions and was in a physical condition normally associated with breeding, but was not seen mate-guarding or competing with other males (Heidi Ahonen, pers. com). With the exception of males at Liguanea and Nicholas Baudin, repeated haulouts at the same colony generally support our assumption but further tracking (particularly during breeding episodes) is required to confirm.

Both [29] and [28] propose that the lack of post-breeding dispersal of male Australian and New Zealand fur seals may relate to increased selective benefits incurred by a) acquiring knowledge of breeding areas throughout the year and b) establishing themselves within the adult male hierarchy at each colony. Additionally, if there is plasticity in the timing of breeding events, there may also be physiological costs to misjudging its onset. Adult male otariids must gain considerable mass prior to breeding in order to compete successfully for territories and females, though accumulating fat is likely to have associated storage and transport costs [88]. Australian sea lion males are confronted with prolonged, aseasonal breeding events that occur asynchronously between colonies. Accordingly they may choose to forage within an area that provides regular access to colonies with breeding schedules that are familiar and can be continually monitored, rather than risk moving to colonies whose breeding chronology is unknown. Physiological costs could also be minimized by accurately predicting the onset of breeding at a colony and delaying fat deposition as long as possible. We propose that the general lack of seasonal dispersal by adult male Australian sea lions is driven by the unique reproductive cycle of adult females and the need to predict the onset of breeding at colonies.

## Conclusions

We present evidence that the foraging behaviour of adult male Australian sea lions is not affected by seasonal changes in oceanographic conditions. Rather, unlike pelagic predators that rely on dynamic environmental features to locate prey, benthically-foraging adult male Australian sea lions may focus on static features that act as predictable prey aggregation sites. Our study contrasts current general assumptions that male otariid life history strategies should result in greater dispersal [23], with adult male Australian sea lions displaying central place foraging behaviour similar to Australian and New Zealand fur seal males [28], [88] and a high degree of fidelity to foraging locations.

In light of extremely low levels of adult female migration and what appears to be a general lack of adult male movement between colonies, the presumption that sufficient male-mediated geneflow occurs to obviate the risks of inbreeding may also be inappropriate. Future work should focus along two mutually non-exclusive lines 1) fully characterising the extent of individual male variability in foraging behaviour both within and between colonies, particularly in relation to breeding cycles of colonies, and 2) determining whether aseasonal foraging behaviour and an apparent lack of dispersal translates into reduced male-mediated geneflow.

## Supporting Information

Table S1 **Core (UD-50) and home range (UD-95) estimates for individual adult male Australian sea lions tracked along the South Australian coast between November 2009 and May 2010.** Males that travelled the furthest during foraging trips had the largest ARS, core and home range estimates. Conversely, the male at Seal Slide occupied the smallest mean home range utilizing less than 140 km^2^ of at-sea habitat.
(DOCX)

Table S2 **The top four Generalised Additive Mixed Models (estimated using δAIC) which best fitted ARS patch size, UD-50 and UD-95 estimates for adult male Australian sea lions tracked along the South Australian coast.** Dynamic and static environmental parameters failed to significantly explain variation in ARS patch size of any male. Similarly, core and home ranges for three males were unrelated to any estimated parameter. For the remaining males, the only dynamic environmental parameters retained in any model were temperature and salinity at the benthos (significant results highlighted in bold).
(DOCX)

Table S3 **Cox Proportional Hazard models fitted to adult male Australian sea lion data, using First Passage Time (FPT) as the independent variable** ***sensu*** **Freitas et al. (2008).** FPT variability of three individuals were most parsimoniously explained by a null model. Exponential coefficient values greater than +1 suggest an aversion to the associated environmental parameter. Thus, males at West Island, Liguanea and West Waldegrave stopped foraging in the presence of increasing benthic temperature and slope, respectively.
(DOCX)

Table S4 **Seasonal Autoregressive Integrated Moving Average (SARIMA) model results for stable isotope ratios of carbon (δ^13^C) and nitrogen (δ^15^N) from the whiskers of seven adult male Australian sea lions instrumented with CTD-SRDL transmitters along the South Australian coastline.** Models are of the form ARIMA(p,d,q) that identify the order of autoregressive (AR) correlation (p), the differencing required for stationarity (d) and the moving average (MA) order (q). Considering stable isotope ratios as a time series, when a seasonal pattern is observed then a seasonal model is added to the ARIMA of the form (P,D,Q), with P, D and Q reflecting the order of AR, differencing and MA component of the seasonal pattern. The number to the right of the seasonal model indicates the lag (in months) of the seasonal term – thus, ‘12’ indicates a seasonal periodicity of 12 months. Ljung-Box Q statistics and associated P values reflect the degree of autocorrelation in SARIMA model residuals, analogous to testing for patterns in the residuals of regression models. Seasonal AR(1) patterns were detected in all ^13^C and three ^15^N isotopic time series. ‘-’ denotes models could not be fitted, or were insignificant. Note, that while seasonal patterns may be detected, the model does not reflect the biological significance of these patterns. For example, although models from the western colonies (i.e West Island and Nicholas Baudin) showed significant patterns with an almost annual cycle, the visualisation of isotope ratios in Figure 4 places the magnitudes of these oscillations in context with similarly significant models from the eastern study sites (Seal Bay and Seal Slide).
(DOCX)
