# Supplementary material for: Steady as He Goes: At-Sea Movement of Adult Male Australian Sea Lions in a Dynamic Marine Environment
Source: PLoS One. 2013 Sep 25;8(9):e74348. doi: 10.1371/journal.pone.0074348 (PMC3783424; doi:10.1371/journal.pone.0074348)
Supplement: Table S1 — Core (UD-50) and home range (UD-95) estimates for individual adult male Australian sea lions tracked along the South Australian coast between November 2009 and May 2010. Males that travelled the furthest during foraging trips had the largest ARS, core and home range estimates. Conversely, the male at Seal Slide occupied the smallest mean home range utilizing less than 140 km2 of at-sea habitat. (DOCX) [file pone.0074348.s001.docx]

**Table S1. Core (UD-50) and home range (UD-95) estimates for individual adult male Australian sea lions tracked along the South Australian coast between November 2009 and May 2010.**  Males that travelled the furthest during foraging trips had the largest ARS, core and home range estimates. Conversely, the male at Seal Slide occupied the smallest mean home range utilizing less than 140km^2^ of at-sea habitat.

| **Animal ID** | **N** | **Utilisation distribution (km^2^)** | | | | **ARS patch size (km^2^)** | |
| --- | --- | --- | --- | --- | --- | --- | --- |
|  | **(trips)** | **50%** | **range** | **95%** | **range** | **mean** | **range** |
|  |  |  |  |  |  |  |  |
| **West Is.** | 19 | 212.6 (89.2) | 63.9-353.9 | 1017.7 (306.5) | 550.3-1626.1 | 17.4 (13.2) | 1.1-49.9 |
| **Nicholas Baudin** | 29 | 67.3 (53.2) | 15.5-205.3 | 421.4 (222.0) | 156.8-997.6 | 10.4 (4.6) | 1 - 22.3 |
| **West Waldegrave** | 42 | 46.4 (32.4) | 12.5-148.9 | 240.7 (147.6) | 68.7-672.6 | 4.3 (3.3) | 0.1-13.5 |
| **Price Is.** | 33 | 40.4 (34.5) | 6.2-136 | 158.8 (126.4) | 29.1-519.9 | 3.1 (2.2) | 0.3-7.6 |
| **Liguanea** | 25 | 85.2 (114.2) | 10.1-463.4 | 376.4 (435.6) | 32.9-1786.3 | 6.4 (4.8) | 0.1-15.5 |
| **Seal Bay** | 23 | 143 (76.2) | 57.7-359.4 | 849.5 (376.1) | 439.6-1977.8 | 11 (5.1) | 2-19.9 |
| **Seal Slide** | 27 | 32.7 (25.1) | 7.3-99.1 | 139.1 (97.0) | 21.8-412.9 | 2.9 (3.3) | 1-15.3 |
|  |  |  |  |  |  |  |  |
