# Supplementary material for: Steady as He Goes: At-Sea Movement of Adult Male Australian Sea Lions in a Dynamic Marine Environment
Source: PLoS One. 2013 Sep 25;8(9):e74348. doi: 10.1371/journal.pone.0074348 (PMC3783424; doi:10.1371/journal.pone.0074348)
Supplement: Table S2 — The top four Generalised Additive Mixed Models (estimated using δAIC) which best fitted ARS patch size, UD-50 and UD-95 estimates for adult male Australian sea lions tracked along the South Australian coast. Dynamic and static environmental parameters failed to significantly explain variation in ARS patch size of any male. Similarly, core and home ranges for three males were unrelated to any estimated parameter. For the remaining males, the only dynamic environmental parameters retained in any model were temperature and salinity at the benthos (significant results highlighted in bold). (DOCX) [file pone.0074348.s002.docx]

**Table S2. The top four Generalised Additive Mixed Models (estimated using dAIC) which best fitted ARS patch size, UD-50 and UD-95 estimates for adult male Australian sea lions tracked along the South Australian coast.** Dynamic and static environmental parameters failed to significantly explain variation in ARS patch size of any male. Similarly, core and home ranges for three males were unrelated to any estimated parameter. For the remaining males, the only dynamic environmental parameters retained in any model were temperature and salinity at the benthos (significant results highlighted in bold).
