# Supplementary material for: Steady as He Goes: At-Sea Movement of Adult Male Australian Sea Lions in a Dynamic Marine Environment
Source: PLoS One. 2013 Sep 25;8(9):e74348. doi: 10.1371/journal.pone.0074348 (PMC3783424; doi:10.1371/journal.pone.0074348)
Supplement: Table S3 — Cox Proportional Hazard models fitted to adult male Australian sea lion data, using First Passage Time (FPT) as the independent variable sensu Freitas et al. (2008). FPT variability of three individuals were most parsimoniously explained by a null model. Exponential coefficient values greater than +1 suggest an aversion to the associated environmental parameter. Thus, males at West Island, Liguanea and West Waldegrave stopped foraging in the presence of increasing benthic temperature and slope, respectively. (DOCX) [file pone.0074348.s003.docx]

**Table S3. Cox Proportional Hazard models fitted to adult male Australian sea lion data, using First Passage Time (FPT) as the independent variable *sensu* Freitas et al. (2008).** FPT variability of three individuals were most parsimoniously explained by a null model. Exponential coefficient values greater than +1 suggest an aversion to the associated environmental parameter. Thus, males at West Island, Liguanea and West Waldegrave stopped foraging in the presence of increasing benthic temperature and slope, respectively.

|  |  | **exp coef (+/- SE coeff, P)** | |  |  | **Random (var)** |
| --- | --- | --- | --- | --- | --- | --- |
|  | **bathymetry** | **benthic salinity** | **slope** | **benthic temp** | **month** | **Trip ID** |
|  |  |  |  |  |  |  |
| **West Is** |  |  |  | **29.96 (1.41, P<0.05)** |  | 0.89 |
| **Nicholas Baudin** | *NULL MODEL (Surv(FPT)~1\|TripID)* | | | | | |
| **West Waldegrave** | **1.1 (0.04, P<0.05)** |  |  |  | **1.1 (0.051, P<0.05)** | 0.03 |
| **Liguanea** |  |  | **134.2 (2.52, P<0.05)** |  |  | 0.03 |
| **Price Is.** | *NULL MODEL (Surv(FPT)~1\|TripID)* | | | | | |
| **Seal Bay** | *NULL MODEL (Surv(FPT)~1\|TripID)* | | | | | |
