# Supplementary material for: Steady as He Goes: At-Sea Movement of Adult Male Australian Sea Lions in a Dynamic Marine Environment
Source: PLoS One. 2013 Sep 25;8(9):e74348. doi: 10.1371/journal.pone.0074348 (PMC3783424; doi:10.1371/journal.pone.0074348)
Supplement: Table S4 — Seasonal Autoregressive Integrated Moving Average (SARIMA) model results for stable isotope ratios of carbon (δ13C) and nitrogen (δ15N) from the whiskers of seven adult male Australian sea lions instrumented with CTD-SRDL transmitters along the South Australian coastline. Models are of the form ARIMA(p,d,q) that identify the order of autoregressive (AR) correlation (p), the differencing required for stationarity (d) and the moving average (MA) order (q). Considering stable isotope ratios as a time series, when a seasonal pattern is observed then a seasonal model is added to the ARIMA of the form (P,D,Q), with P, D and Q reflecting the order of AR, differencing and MA component of the seasonal pattern. The number to the right of the seasonal model indicates the lag (in months) of the seasonal term – thus, ‘12’ indicates a seasonal periodicity of 12 months. Ljung-Box Q statistics and associated P values reflect the degree of autocorrelation in SARIMA model residuals, analogous to testing for patterns in the residuals of regression models. Seasonal AR(1) patterns were detected in all 13C and three 15N isotopic time series. ‘-’ denotes models could not be fitted, or were insignificant. Note, that while seasonal patterns may be detected, the model does not reflect the biological significance of these patterns. For example, although models from the western colonies (i.e West Island and Nicholas Baudin) showed significant patterns with an almost annual cycle, the visualisation of isotope ratios in Figure 4 places the magnitudes of these oscillations in context with similarly significant models from the eastern study sites (Seal Bay and Seal Slide). (DOCX) [file pone.0074348.s004.docx]

Table S4. Seasonal Autoregressive Integrated Moving Average (SARIMA) model results for stable isotope ratios of carbon (δ^13^C) and nitrogen (δ^15^N) from the whiskers of seven adult male Australian sea lions instrumented with CTD-SRDL transmitters along the South Australian coastline. Models are of the form ARIMA(p,d,q) that identify the order of autoregressive (AR) correlation (p), the differencing required for stationarity (d) and the moving average (MA) order (q). Considering stable isotope ratios as a time series, when a seasonal pattern is observed then a seasonal model is added to the ARIMA of the form (P,D,Q), with P, D and Q reflecting the order of AR, differencing and MA component of the seasonal pattern. The number to the right of the seasonal model indicates the lag (in months) of the seasonal term – thus, ‘12’ indicates a seasonal periodicity of 12 months. Ljung-Box Q statistics and associated P values reflect the degree of autocorrelation in SARIMA model residuals, analogous to testing for patterns in the residuals of regression models. Seasonal AR(1) patterns were detected in all ^13^C and three ^15^N isotopic time series. ‘-‘ denotes models could not be fitted, or were insignificant. Note, that while seasonal patterns may be detected, the model does not reflect the biological significance of these patterns. For example, although models from the western colonies (i.e West Island and Nicholas Baudin) showed significant patterns with an almost annual cycle, the visualisation of isotope ratios in Figure 4 places the magnitudes of these oscillations in context with similarly significant models from the eastern study sites (Seal Bay and Seal Slide).

|  | **δ^13^C** | | | | **δ^15^N** | | | |
| --- | --- | --- | --- | --- | --- | --- | --- | --- |
|  | **SARIMA** | **AIC** | **Ljung-Box Q** | **P** | **SARIMA** | **BIC** | **Ljung-Box Q** | **P** |
| **West Island** | (1,1,0)s(1,1,0)x11 | 36 | 0.02 | 0.87 | - | - | - | - |
| **Nicholas Baudin** | (0,1,0)s(0,1,0)x12 | -37.9 | <0.01 | 0.98 | - | - | - | - |
| **West Waldegrave** | (1,1,0)s(1,1,0)x12 | 30.4 | 0.22 | 0.64 | - | - | - | - |
| **Price Island** | (1,1,0)s(5,0,0)x9 | 0.5 | 0.97 | 0.32 | - | - | - | - |
| **Liguanea** | (1,1,0)s(1,1,0)x10 | 20.9 | 0.33 | 0.56 | (1,1,0)s(1,1,0)x11 | 13.6 | 0.25 | 0.22 |
| **Seal Slide** | (1,1,0)s(1,1,0)x12 | 1.8 | 0.24 | 0.62 | (1,1,0)s(1,1,0)x15 | -5.8 | 0.54 | 0.78 |
| **Seal Bay** | (1,1,0)s(1,1,0)x13 | 29.9 | 0.13 | 0.87 | (1,1,0)s(1,1,0)x8 | -36.4 | 0.18 | 0.94 |
